# Morphological and ultrasonographic characterization of the three zones of supratesticular region of testicular artery in Assaf rams

**DOI:** 10.1038/s41598-022-12243-z

**Published:** 2022-05-18

**Authors:** Mohamed A. A. Hassan, Ramy K. A. Sayed, Mohammed Abdelsabour-Khalaf, Enas A. Abd-Elhafez, L. Anel-Lopez, M. F. Riesco, C. Ortega-Ferrusola, R. Montes-Garrido, M. Neila-Montero, L. Anel, M. Alvarez

**Affiliations:** 1 Department of Anatomy and Embryology, Faculty of Veterinary Medicine, Sohag University, Sohag, 82524 Egypt; 2 Department of Anatomy and Embryology, Faculty of Veterinary Medicine, South Valley University, Qena, 83523 Egypt; 3 Department of Anatomy and Histology, Faculty of Veterinary Medicine, Assiut University, Assiut, 71526 Egypt; 4 ITRA-ULE, INDEGSAL, University of León, 24071 León, Spain; 5 Department of Veterinary Medicine, Surgery and Anatomy, University of León, 24071 León, Spain; 6 Cellular Biology, Department of Molecular Biology, University of León, 24071 León, Spain; 7 Laboratory of Equine Reproduction and Equine Spermatology, Veterinary Teaching Hospital, University of Extremadura, Cáceres, Spain

**Keywords:** Anatomy, Cells, Gonads

## Abstract

To fully understand the histological, morphometrical and heamodynamic variations of different supratesticular artery regions, 20 mature and healthy Assaf rams were examined through ultrasound and morphological studies. The testicular artery images of the spermatic cord as shown by B-mode analysis indicated a tortuous pattern along its course toward the testis, although it tends to be less tortuous close to the inguinal ring. Doppler velocimetric values showed a progressive decline in flow velocity, in addition to pulsatility and vessel resistivity when entering the testis, where there were significant differences in the Doppler indices and velocities among the different regions. The peak systolic velocity, pulsatility index and resistive index were higher in the proximal supratesticular artery region, followed by middle and distal ones, while the end diastolic velocity was higher in the distal supratesticular region. The total arterial blood flow and total arterial blood flow rate reported a progressive and significant increase along the testicular cord until entering the testis. Histological examination revealed presence of vasa vasorum in the tunica adventitia, with their diameter is higher in the proximal supratesticular zone than middle and distal ones. Morphometrically, the thickness of the supratesticular artery wall showed a significant decline downward toward the testis; meanwhile, the outer arterial diameter and inner luminal diameter displayed a significant increase distally. The expression of alpha smooth muscle actin and vimentin was higher in the tunica media of the proximal supratesticular artery zone than in middle and distal ones.

## Introduction

Sperm production and semen quality are affected mainly by testicular hemodynamics, which is the main route for nutrients, hormones, and oxygen supply^1^. The testis consists of a high metabolic tissue rate and any reduction in the testicular blood flow will affect negatively in spermatogenesis and testicular morphology^2^.

Testicular artery is considered the main route for testicular blood supply. It originates directly from the ventral surface of the abdominal aorta, and its diameter is wider in the ram than other medium and small-sized animals^3^. Previous study was performed on the testicular artery of the Egyptian buffalo bull, and reported that the artery includes four parts during its course; abdominal, funicular, marginal, and parenchymal. The abdominal part extends from the origin of the testicular artery at its exit from the abdominal aorta till the internal opening of the inguinal canal, while the funicular part occupies the spermatic cord, which starts from the external opening of the inguinal canal till the proximal extremity of the testis. The marginal part of the testicular artery is the normal continuation of the funicular part, and is located on the epididymal margin of the testis and extends between proximal and distal extremities of the testis. The parenchymal part is formed of group of branches run through the texture of the testicular parenchyma towards the mediastinum testis^4^.

Color pulsed-wave Doppler and B-mode ultrasonography is a non-invasive tool, which was developed to allow the study of anatomical data of blood vessels and to evaluate functional data regarding vascular hemodynamics; including its presence or absence, direction and speed. It is the method of choice for assessing the vascularity of different organs, including the testes^5^. Doppler is widely used in human medicine to evaluate testicular artery blood flow and pathological conditions, which are associated with blood flow alternation^6^. Recently, it has been used for assessment of testicular blood flow in domestic species as camels^7^, stallions^8^, bulls^9^, rams^10^, dogs^11^ and goats^12^. The abovementioned clarity of testicular artery is not used in most studies during characterizing the arterial regions, and it is believed that absence of anatomical description consistency and recent improvements in ultrasound equipment quality may clarify conflicting results detected between studies; specially regional differences in testicular artery hemodynamic parameters^13^.

Recently, three regions in the testicular artery have been used frequently for evaluation of blood flow to the testis in routine clinical procedures and hemodynamic differences among these regions have been reported. These regions are defined as supratesticular (in the spermatic cord), marginal (on the epididymal edge of testis), and intratesticular regions^8,14,15^. The supratesticular region is the most important region of the testicular artery, and it is frequently used in the evaluation of testicular blood flow in rams^10,16^.

Histologically, testicular artery is considered a muscular artery, where its wall consists of three layers or tunics. The tunica intima is formed of endothelium, thin layer of loose connective tissue and prominent internal elastic lamina. The tunica media is consisted mainly of smooth muscle cells, with elastic fibers, elastic lamellae and reticular fibers. The outermost tunica adventitia is constituted of collagen and elastic fibers, and contains a network of unmyelinated autonomic vasomotor nerve fibers^17^.

Because of significant importance of the supratesticular region in evaluation of the testicular vasculature and its extension in the spermatic cord, this study aimed to investigate histological, ultrasonographic, and morphometrical characteristics of different parts of this region (proximal, middle, and distal) in Assaf rams for further standardization of the measurement site of the routine clinical procedure for evaluation of testicular blood flow, since a few centimeters can affect greatly in the hemodynamic parameters^13,18^.

## Material and methods

### Animals and sample preparation

A total of 20 mature and healthy Assaf rams (2–7 years old) with no history of any previous reproductive disorders were used during the breeding season. The rams were kept at Centro de Selección y Reproducción Animal (CENSYRA), León, Spain. The current study was performed in compliance with the recommendations in the ARRIVE guidelines according to the Guidelines of the European Union Council (86/609/EU, modified by 2010/62/EU), following Spanish regulations (RD/1201/2005, abrogated by RD/2013) for the use of laboratory animals. All experimental protocols and procedures were approved by the institutional animal care and use committee at the University of León (Spain) (ETICA-ULE-034-2020). Scrotal circumference was measured just before ultrasonographic assessment. After slaughtering, testes and spermatic cords were directly taken by inguino scrotal incision and were washed by normal saline to remove blood. The testicular artery in the supratesticular region was dissected, and was subdivided into three parts; 1) proximal of testicular artery near the inguinal ring, 2) middle of testicular artery in the middle way of the spermatic cord, 3) distal of testicular artery at the distal end of the spermatic cord, proximal to the testis.

### Ultrasonographic assessment

The ultrasound equipment used in this study is an EXApad® (IMV Imaging, Angoulême, France) with two different probes: 5–10 MHz linear transducer (LR760V®) and 7.5–15 MHz linear transducer (LC1038V®). All ultrasound examinations were performed using the same operator to evade variation. The rams were restrained with an injection of tranquilization with Xylazine 20 mg (Rompun®, Bayer, Leverkusen, Germany; 0,05 mg/kg i.m.). To avoid the presence of air spaces, the wool of the scrotum was shaved and washed with water, followed by application of alcohol. Finally, the transducer was covered with a plenteous amount of gel to favor ultrasonographic imaging.

#### B-mode ultrasonographic evaluation

This imaging modality was carried out to identify different anatomical structures of the testis and also to diagnose probable pathologic conditions. At least three separated longitudinal and transverse testicular images were recorded, and the length, width and thickness of the testis were measured using electronic calipers. The testis volume (TV) was calculated through the formula for measuring an oblate ellipsoid volume: 4/3π × A × B × C^19^, where Total Testicular Volume (TTV) is the sum of the both testicles volume. At least three separated images of supratesticular region of testicular artery were described in three locations; (1) near the inguinal ring (proximal part), (2) in the mid-way of the spermatic cord (middle part), (3) At the distal part of the spermatic cord, proximal to the testis (distal part). The testicular artery diameter at each part was analyzed.

#### Color flow doppler (CFD) and pulsed-wave color doppler (PWD) evaluation

Doppler analysis was performed firstly by the B-mode and CFD modality to recognize the arterial vessel. Therefore, PWD was carried out to quantify the blood flow velocity inside the vessel. For distinguishing the artery from the vein using Doppler evaluation, the artery has a wave form on the spectral graph parallel with the arterial pulse, while the venous flow is almost stable, without a pulse. The used angle between the long longitudinal vessel axis and Doppler beam was between 30 and 40 in the blood flow direction. The gate of the Doppler was kept steady at 1 mm.

After spectral pattern appearance of the testicular artery, the Doppler velocites (cm/s) including End Diastolic Velocity (EDV), Peak Systolic Velocity (PSV), and the Time Average Maximum Velocity (TAMV), as well as Doppler indices including Pulsatility Index (PI): PSV-EDV/TAMV and Resistive Index (RI): PSV-EDV/PSV were studied^20^. Total arterial blood flow (TABF: TAMV x A; where A: πr2 is the cross-sectional area of the testicular artery) and total arterial blood flow rate (TABF rate: TABF/TTV × 100) were also recorded for all studied animals as testicular perfusion indices. Three to five measurements were determined for each parameter in various locations over the testicular artery. The wave forms of the testicular artery are characterized as either resistive or non-resistive. Resistive wave forms are characterized by high variation degree between systolic and diastolic velocity of blood with high values of resistive index, in contrast to non-resistive wave forms. All ultrasound settings including brightness, gains, focus, and contrast were fixed, and used equally for all examinations in order to reduce recording variations.

### Histological examination

For histological analysis, samples from different supratesticular zones of the supratesticular region of the testicular artery were washed with normal saline, and were fixed in Bouin's solution for 24 h. Fixed samples were then dehydrated through passing in an ethanol ascending series, and were cleaned in methyl benzoate and were embedded in paraffin wax. Sections of 5–8 µm thickness were cut and were stained with Harris hematoxylin and Eosin for general structure, Crossmon's trichrome for collagen and muscle fibers, and Wigert's Elastica for identification of elastic fibers^21^. Sections were then examined using an OPTIKA B-293 microscope, and digital images were acquired by OPTIKA C-B10 camera and OPTIKA PRO View software.

### Immunohistochemical analysis

Paraffin sections from different supratesticular zones of the testicular artery in the supratesticular region were prepared for further immunohistochemical studies. After deparaffinization using xylene, sections were rehydrated in ethanol and were washed in distilled water. Specimens were heated in sodium citrate buffer (0.01 M, pH 6.0) for 15 min in a microwave oven in order to increase epitope exposure. Following that, samples were cooled, and were washed with phosphate buffered saline before being blocked using 10% bovine serum albumin (BSA) at room temperature for 1 h. Sections were then incubated overnight at 4 °C using mouse monoclonal anti-human α-smooth muscle actin (α-SMA) (1:300, Dako, Hamburg, Germany, M0851) and mouse monoclonal anti-vimentin (1:400, Thermo Fisher Scientific, USA, MS-129-R7). Primary antibodies were visualized using an SABC Kit Elite and 0.05% 3, 3-diaminobenzidine tetrachloride (Sigma) in PBS. The slides were finally counterstained with hematoxylin and were mounted and covered with cover slips. 1% BSA was used instead of primary antibody to examine antibody specificity.

### Morphometrical analysis

Various morphometrical parameters of the testicular artery including thickness of the arterial wall and its different layers, outer arterial diameter, inner luminal diameter, number of smooth muscle cell rows of tunica media, and diameter of vasa vasorum were measured on images of light microscopy using ImageJ version 1.53 processing software.

### Statistical analysis

Statistical analyses of the obtained data were performed using GraphPad Prism software version 8.4.0 (GraphPad Software, San Diego, California USA). Moreover, one-way ANOVA with a Tukey’s post hoc test was used for comparing significance between studied parts. Differences were considered significant when *P* < 0.05. Data were presented as mean ± *SEM*.

## Results

### Ultrasound examination

#### B-mode analysis

B-mode analysis for testicular measurements was conducted in all studied animals. The summary of the descriptive statistics for both testicular and scrotal measurements are shown in (Table 1). B-mode, continuous wave Doppler, and pulsed wave Doppler analysis were performed in 13 rams, and in all previously described zones of the supratesticular region of the testicular artery. Doppler analysis for the testicular artery marginal zone was applied; however, the equipment can not recognize this region because of its decreased blood velocity and low PRF (pulse repetition frequency). The mean time of analysis of all zones of the two testicles was approximately 30 min.

**Table 1:** Measurements (mean ± SEM) of testis, epididymis, and pampiniform plexus with B-Mode ultrasonography.

Scrotal circumference	Testis				
	Width (cm)	Thickness (cm)	Length (cm)	Volume (cm^3^)	TTV (cm^3^)
33.6 ± 0.4	6.5 ± 0.1	5.8 ± 0.1	8.3 ± 0.1	170.9 ± 5.1	341.8 ± 14.1

Epididymis			Pampiniform plexus		
Width (cm)	Thickness (cm)	Length (cm)	Length (cm)	Width (cm)	Area (cm^2^)
2.5 ± 0.1	3.7 ± 0.1	3.7 ± 0.04	3.6 ± 0.1	2.9 ± 0.1	10.5 ± 0.4

**Figure 1:**
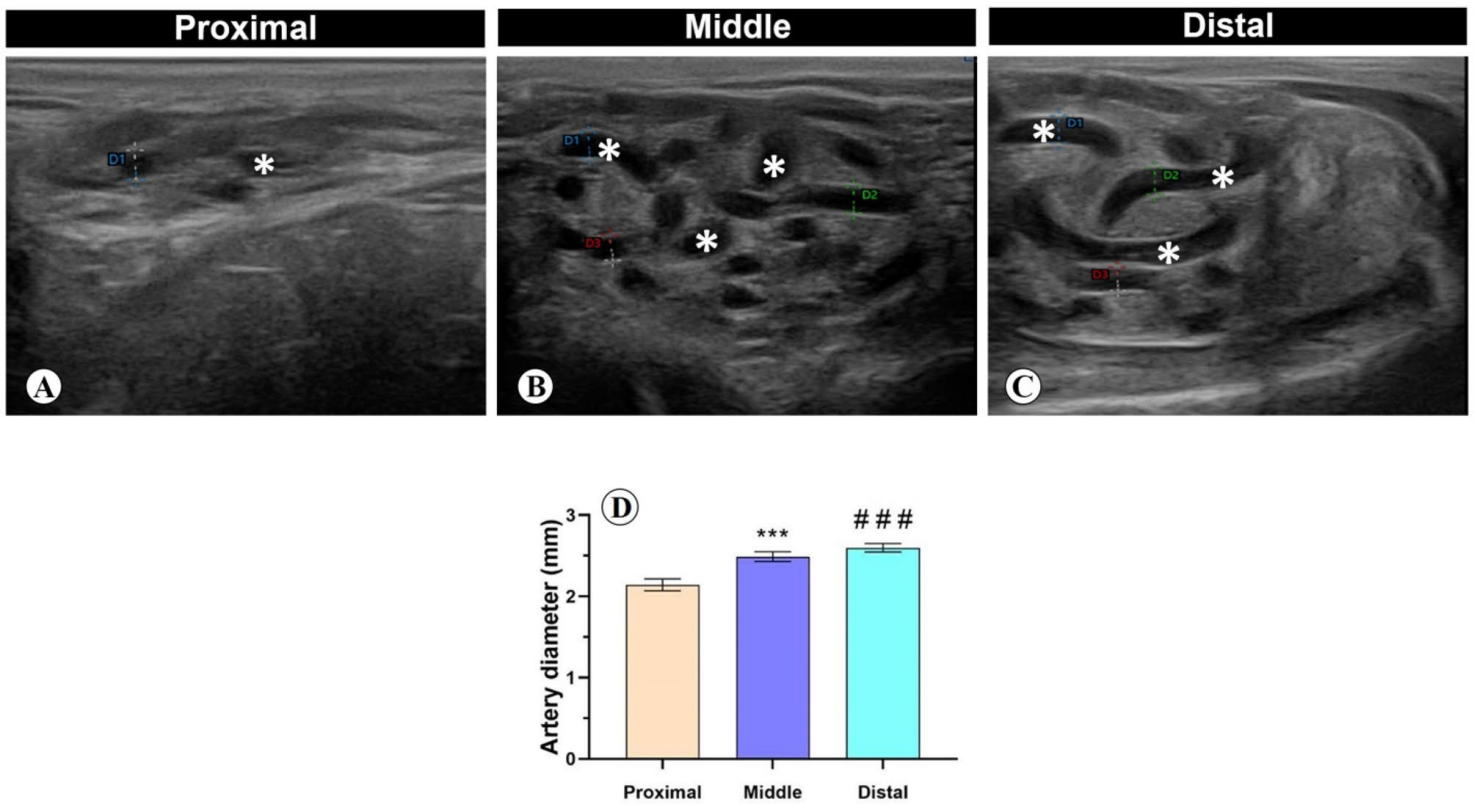
Cross section of the spermatic cord with B-mode ultrasonography. (**A**) Proximal, (**B**) middle, and (**C**) distal supratesticular zones of the testicular artery (stars). (**D**) Testicular artery diameter calculated by electronic caliper with B-mode ultrasonography in proximal, middle and distal supratesticular zones. ***p < 0.001 Middle versus Proximal zone; ^###^p < 0.001 Distal versus Proximal zone.

The spermatic cord appeared as a non-echoic circular zone with a black colour, surrounded by whiter hyperechoic regions, and the colour Doppler of the pampiniform plexus was detected as large spots with varying degree of orange and blue colouration during scanning (Fig. 1A–C).

The diameter of the testicular artery revealed an increase from proximal to distal zones of the supratesticular region, where there was a significant raise in the diameter of distal and middle zones compared with proximal one, meanwhile the diameter values between middle and distal zones were not significant. The diameter of the proximal, middle and distal zones of supratesticular region of the testicular artery was 2.1 ± 0.1 mm, 2.5 ± 0.1 mm, and 2.6 ± 0.1 µm, respectively (Fig. 1D).

#### Continuous wave doppler (CFD) and pulsed wave doppler (PWD)

**Figure 2:**
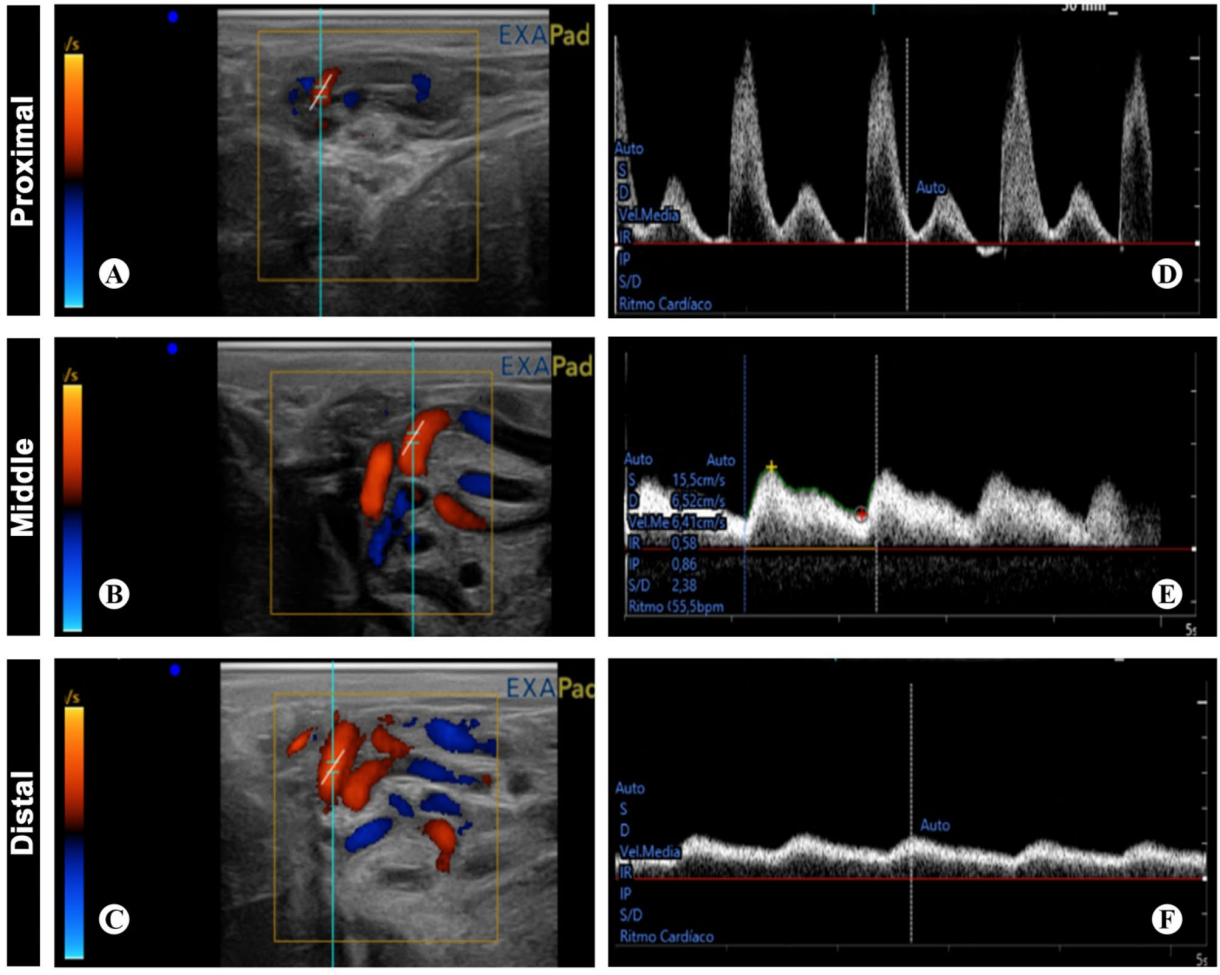
Cross section of the spermatic cord with color and pulse Doppler ultrasound. (**A**,**D**) proximal, (**B**,**E**) middle, and (**C**,**F**) distal supratesticular zones of the testicular artery illustrating Doppler parameters calculated by the ultrasound equipment’s software.

Doppler wave morphology showed variation among studied zones. The proximal zone of the testicular artery demonstrated a high resistive biphasic wave form pattern with high sharp systolic peak, low EDV and narrow spectral window. The middle zone depicted intermediate resistivity that is associated with biphasic wave form pattern, while the distal zone recorded the lowest resistivity with monophasic wave form pattern, low blunt systolic peak, high EDV and wide spectral window (Fig. 2A–F).

**Figure 3:**
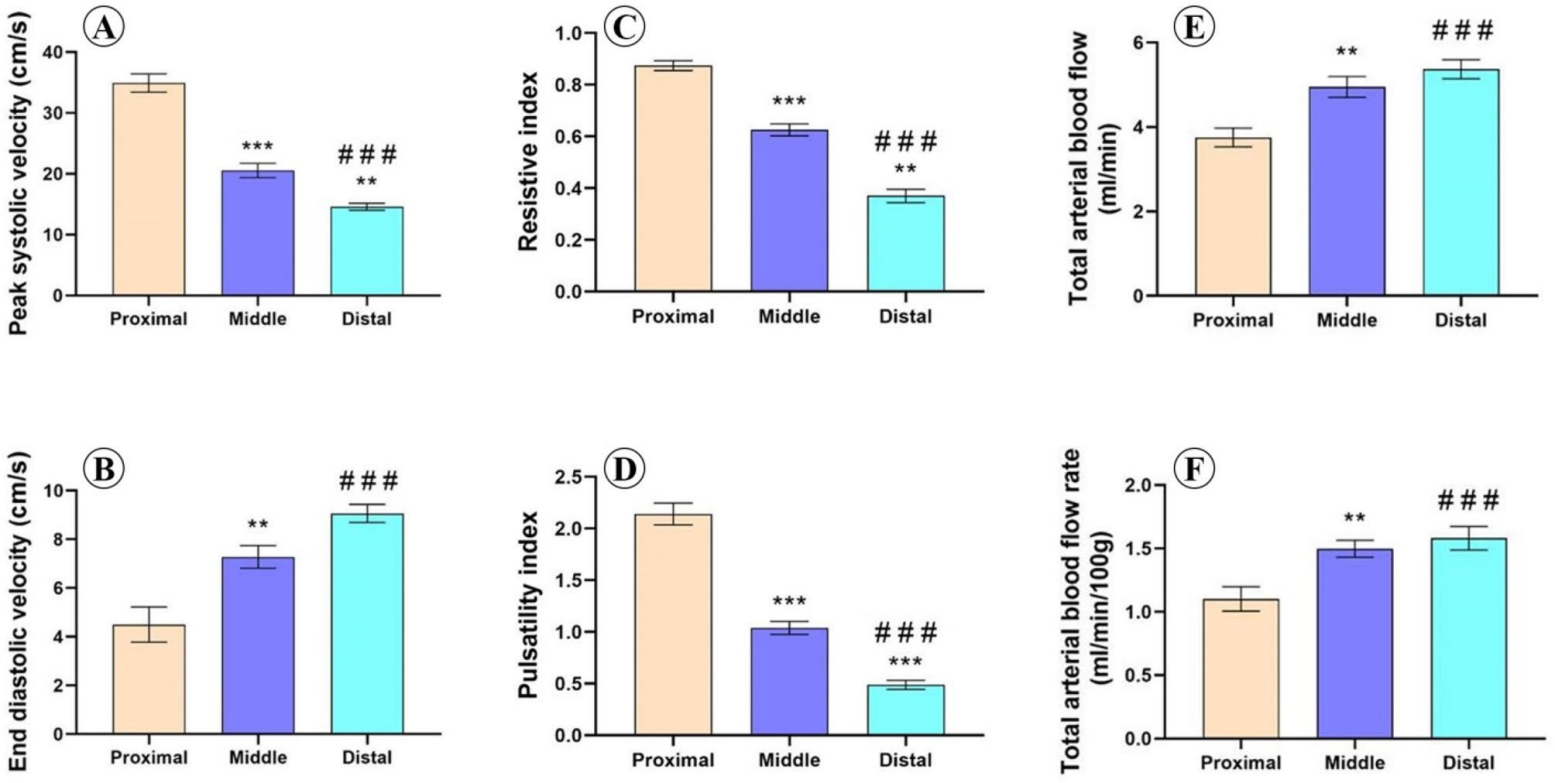
Doppler parameters measured in proximal, middle and distal supratesticular zones in the supratesticular region along the testicular cord. (**A**) Peak systolic velocity (cm/s); (**B**) End diastolic velocity (cm/s); (**C**) Resistive index; (**D**) Pulsatility index; (**E**) Total arterial blood flow (ml/min) and (**F**) Total arterial blood flow rate (ml/min/100 g of testicular tissue). **p < 0.01 and ***p < 0.001 versus proximal zone; ^###^p < .001 versus middle zone.

Doppler velocimetric values in various zones demonstrated a progressive decline in flow velocity, in addition to pulsatility and vessel resistivity when entering the testis, where there were significant differences in the Doppler indices and velocities between the different zones. The PSV was 34.9 ± 1.5 cm/s, 20.6 ± 1.2 cm/s, and 14.6 ± 0.6 cm/s, in proximal, middle, and distal zones of supratesticular region of testicular artery, respectively. Moreover, the EDV was 4.5 ± 0.7 cm/s in the proximal supratesticular zone, 7.3 ± 0.5 cm/s in the middle supratesticular zone, and 0.1 ± 0.4 cm/s in the distal supratesticular zone (Fig. 3A,B).

Resistive Index (RI) was 0.9 ± 0.02, 0.6 ± 0.02, and 0.4 ± 0.03, in proximal, middle, and distal zones of supratesticular region of testicular artery, respectively. Furthermore, PI was 2.1 ± 0.1 in the proximal supratesticular zone, 1 ± 0.1 cm/s in the middle supratesticular zone, and 0.5 ± 0.04 cm/s in the distal supratesticular zone (Fig. 3C,D).

The total testicular blood perfusion represented by TABF and TABF rate reported a progressive and significant increase along the spermatic cord until entering the testes. TABF was 3.8 ± 0.2 ml/min, 4.95 ± 0.3 ml/min, and 5.4 ± 0.2 ml/min in proximal, middle, and distal supratesticular zones of testicular artery, respectively. TABF rate was 1.1 ± 0.1 of testicular tissue in the proximal supratesticular zone, 1.5 ± 0.1 of testicular tissue in the middle supratesticular zone, and 1.6 ± 0.1 of testicular tissue in the distal supratesticular zone (Fig. 3E,F).

### Light microscopy and morphometry

**Figure 4:**
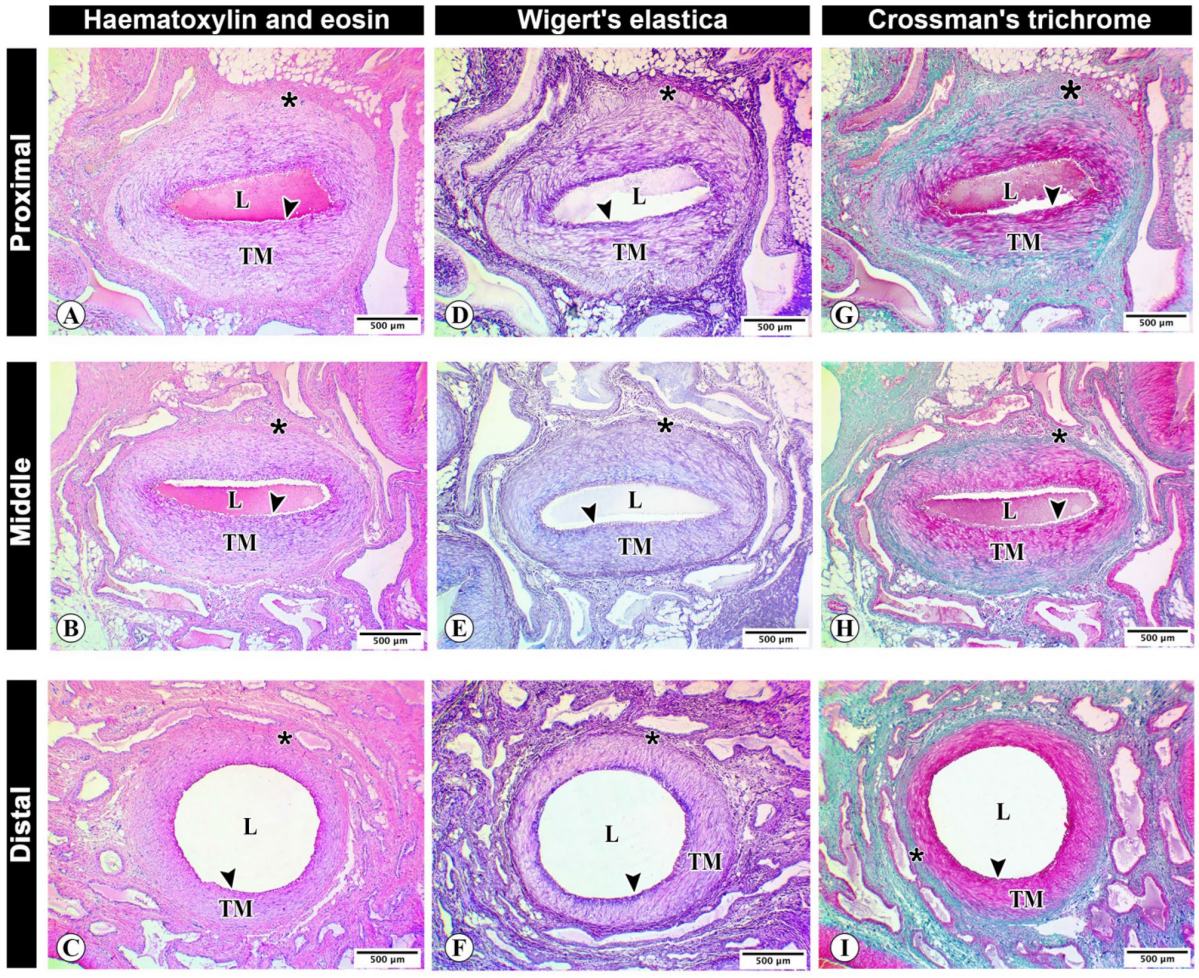
Histological architecture of the testicular artery wall. Photomicrographs of cross sections of proximal (**A**,**D**,**G**), middle (**B**,**E**,**H**), and distal (**C**,**F**,**I**) supratesticular zones of the testicular artery in the supratesticular region stained by H&E (**A**–**C**), Weigert’s elastic (**D**–**F**) and Crossmon’s trichrome (**G**–**I**) stains showing lumen of the testicular artery (**L**), tunica intima (arrowheads), tunica media (TM), and tunica adventitia (asterisks) with deposition of collagenous tissues (stained green with Crosson’s trichrome). Bar 500 µm.

**Figure 5:**
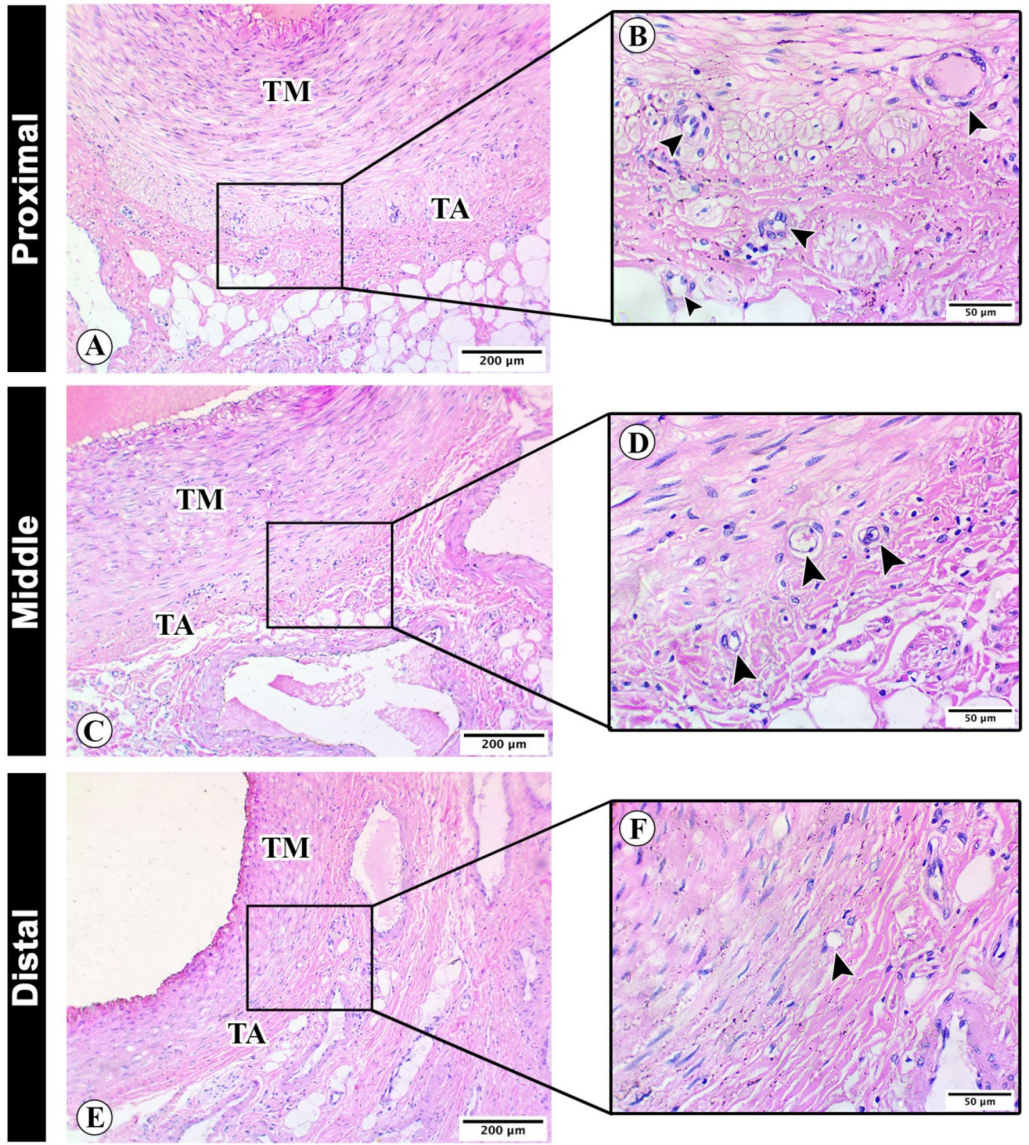
Histological analysis of the testicular artery tunics. Photomicrographs of cross sections of proximal (**A**,**B**), middle (**C**,**D**), and distal (**E**,**F**) supratesticular zones of the testicular artery in the supratesticular region stained by H&E showing vasa vasorum (arrowheads). tunica media (TM) and tunica adventitia (TA). (**A**,**C**,**E**): bar 200 µm and (**B**,**D**,**F**): bar 50 µm.

Histological examination of proximal, middle and distal supratesticular zones of testicular artery revealed the three distinct layers or tunica. The collagenous tissue infiltrations in tunica adventitia showed a considerable decline toward the distal supratesticular region (Fig. 4A–I). The blood vessels were more detectable in the proximal supratesticular zone than in the middle and distal ones (Fig. 5A–F).

**Figure 6:**
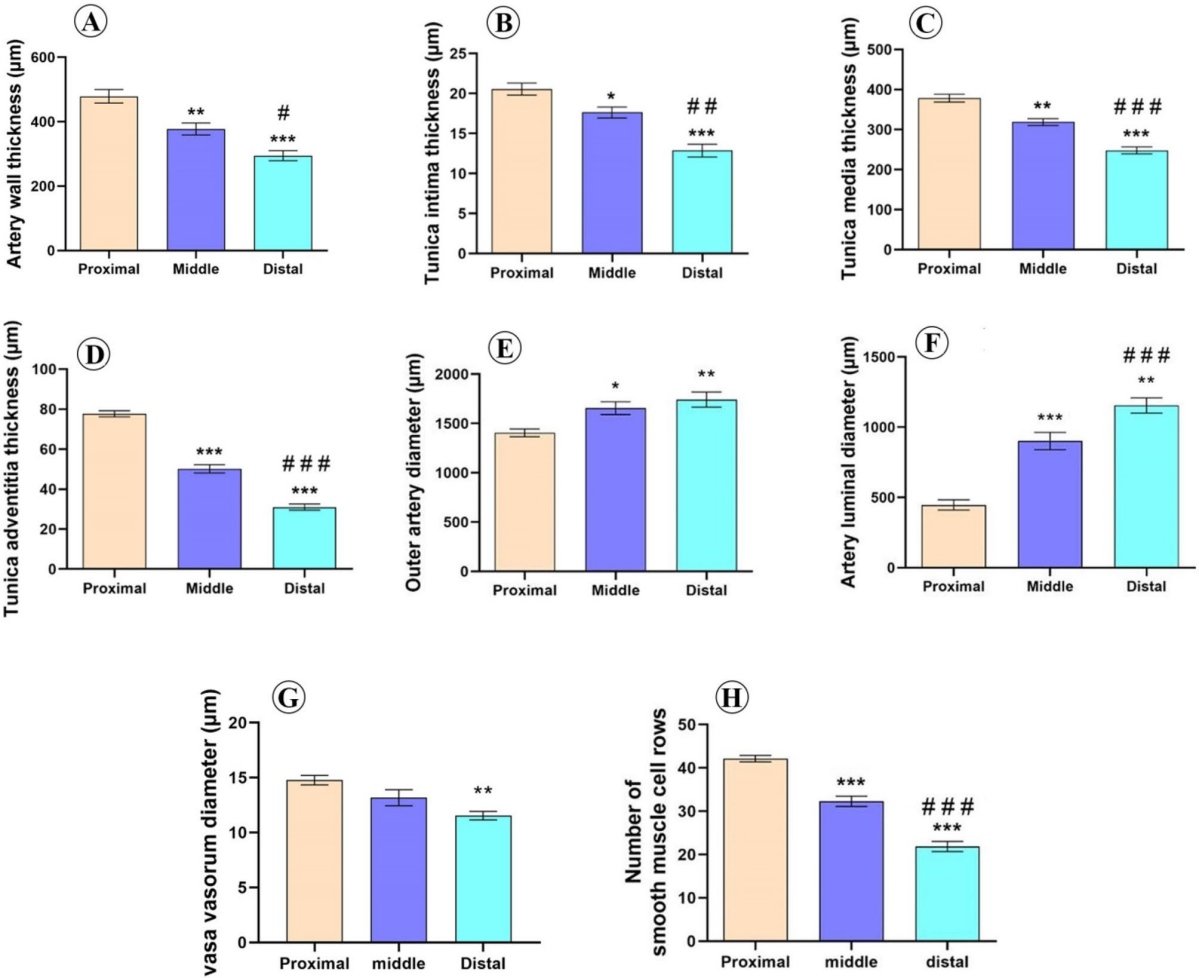
Morphometrical analysis of the testicular wall in proximal, middle, and distal supratesticular zones of the testicular artery in the supratesticular region. (**A**) Artery wall thickness; (**B**) Tunica intima thickness; (**C**) Tunica media thickness; (**D**) Tunica adventitia thickness; (**E**) Outer arterial diameter; (**F**) Inner luminal diameter; (**G**) Vasa vasorum diameter; (**H**) Number of smooth muscle cell rows of tunica media. *p < 0.05, **p < 0.01 and ***p < 0.001 versus proximal zone; ^#^p < 0.05, ^##^p < 0.01 and ^###^p < 0.001 versus middle zone.

Morphometrical analysis of different zones of supratesticular region of testicular artery revealed that the thickness of the arterial wall was significantly decreased toward the distal zone. The wall thickness of the proximal, middle and distal supratesticular zones of testicular artery was 478.9 ± 21.1 µm, 377.1 ± 18.5 µm, and 294.1 ± 15.6 µm, respectively (Fig. 6A).

Thicknesses of tunica intima, tunica media and tunica adventitia displayed also significant reductions distally. These layers measured 20.6 ± 0.8 µm, 378.2 ± 9.8 µm, and 77.7 ± 1.6 µm, respectively in the proximal supratesticular zone, while they measured 17.6 ± 0.7 µm, 318.8 ± 8.4 µm, and 50.2 ± 2.1 µm, respectively in the middle zone, as well as 12.9 ± 0.8 µm, 247.9 ± 8.7 µm, and 31 ± 1.5 µm, respectively in the distal zone (Fig. 6B–D).

Outer arterial diameter and inner luminal diameter were significantly increased downward toward the testis. The outer arterial diameter was 1405 ± 39.5 µm, 1655 ± 64.9 µm, and 1743 ± 76.9 µm, in proximal, middle, and distal supratesticular zones of testicular artery respectively (Fig. 6E). Moreover, the inner luminal diameter was 446.9 ± 36.5 µm in the proximal supratesticular zone, 901 ± 62.2 µm in the middle supratesticular zone, and 1155 ± 54.2 µm in the distal supratesticular zone (Fig. 6F).

The vasa vasorum diameter showed a reduction distally (Fig. 6G). Tunica media displayed a significant decline in the number of smooth muscle cell rows distally toward the testis. In the proximal supratesticular zone, tunica media was formed of 42.1 ± 0.7 rows of smooth muscle cells, while middle and distal zones were constituted of 32.3 ± 1.2 rows and 21.9 ± 1.2 rows, respectively (Fig. 6H).

### Immunohistochemistry

**Figure 7:**
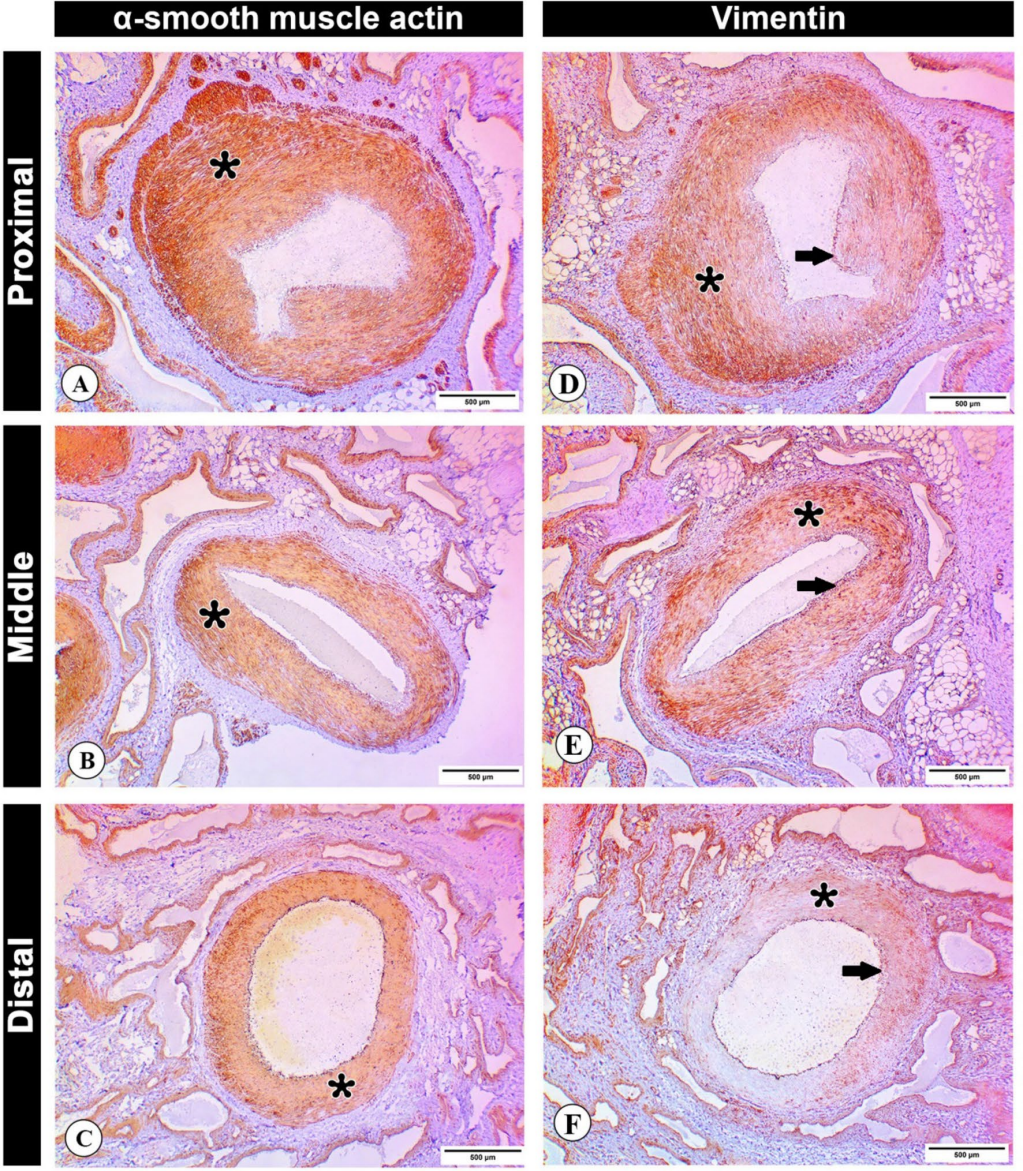
Immunohistochemical analysis of the testicular artery wall. (**A**–**C**) Photomicrographs of cross sections of proximal (**A**), middle (**B**), and distal (**C**) supratesticular zones of the testicular artery in the supratesticular region showing an immunohistochemical expression of α-SMA in tunica media (asterisks). (**D**–**F**) Photomicrographs of cross sections of proximal (**D**), middle (**E**), and distal (**F**) supratesticular zones of the testicular artery depicting an immunohistochemical expression of vimentin in tunica media (asterisks). Note the expression of vimentin in the endothelial cells of the tunica intima (arrows). Bar 500 µm.

α-SMA expression in the arterial wall was detected in tunica media of proximal, middle and distal zones of supratesticular region of testicular artery; however, of the expression was higher in the proximal zone than in the middle and distal ones (Fig. 7A–C).

Layers of the supratesticular region of testicular artery in the proximal and middle zones revealed higher expression of vimentin, while less immunostaining was observed in the distal zone. Moreover, the expression of vimentin was detected in endothelial cells of the tunica intima (Fig. 7D–F).

## Discussion

Testis consists of high active metabolic tissue that is very sensitive to minor disturbance in its nutritive supply. This supply comes mainly via the testicular artery, which has a high flow resistance, as it originates directly from the abdominal aorta, leaving ultimately intratesticular capillary pressure lower than that of other organ, and only slightly higher than the venous pressure^22,23^.

The oxygen concentration in the testis is low^24^, and thus, high metabolic seminiferous tubules tissues get adapted to this low tension of oxygen level environment and low blood perfusion pressure. This situation suggests that any disturbances in blood perfusion and hemodynamics can induce ischemic damage and compromised testicular functions^23^. The investigation of arterial blood flow of male reproductive organs especially in rams has rarely been studied^19^.

Previous studies investigated the measurements in supratesticular region; however, the findings were imprecise about the measurement's exact location. Moreover, the proximal and distal supratesticular zones have not yet been described^19,20,25,26^.

In this study, Doppler analysis was performed for the marginal zone of the testicular artery (on the epididymal edge of testis); however, the ultrasound equipment couldn’t interpret the given Doppler waves because of the small-sized vessel and decreased blood flow velocity in this area. In this concern, the hemodynamic measurements of the testicular artery of the dog at the marginal zone were evaluated using Doppler ultrasonography^13^.

As shown here, right and left testes of Assaf rams revealed no significant difference (*p* > 0.05); and subsequently, the means of both testes were used for other performed analyses. This finding is in agreement with recent studies, where testes develop in a highly symmetrical manner in ram lambs^19^. Similar results were obtained in Barki ram^25^, Dorper ram^19^ and Egyptian fat-tailed rams^10^. Furthermore, the Doppler testicular artery indices were not significantly affected by body weight, age, and pulse rate^25^.

The scrotal circumference values of Assaf rams was 33.63 ± 0.41 cm, with values ranging from 29.8 to 36.3 cm. Batissaco et al.^20^ observed a mean of 32.93 cm in Santa Inês rams aged 1–6 years old. Similar results were reported by Camela et al.^19^, where a mean scrotal circumference of 33.3 cm in Dorper rams with mean age of 16.6 months was reported. Previous study reported the high correlation between ovine and bovine scrotal circumference with the sperm production and normal sperm percentage^27^.

Testicular volume is a principal sperm production indicator, where the seminiferous tubules exemplify the major testicular constituent^26,28–30^. The obtained values of testicular length, width, thickness, and total testicular volume were parallel to those previously reported in rams^19,20^.

By B-mode ultrasonography, the spermatic cord appeared as a non-echoic circular zone with a black colour, surrounded by whiter hyperechoic regions; meanwhile, the colour Doppler of the pampiniform plexus was detected as large spots with varying degree of orange and blue colouration during scanning. These findings were in accordance with those previously described in rams^10,19,25^.

B-mode ultrasonography images revealed significant variations between testicular artery diameters in the spermatic cord at the three studied locations. The diameter progressively increases along its course till entering the testis. This progressive increase in diameter together with an increased tortuosity allow more surface area for heat exchange to maintain the testicular temperature in rams of approximately 4 °C below body temperature^31^.

RI and PI are two haemodynamic indicators measured in the testicular artery that may be potentially correlated to semen quality in stallions^8^, Dogs^32^ and rams^20,26^. Recently, RI and PI have provided useful records for predicting future testicular function and ejaculate quantity of rams^25^.

In this study, the testicular blood flow pattern as measured by colour spectral Doppler ultrasonography revealed significant variations in RI and PI among the three studied zones, where the RI and PI values showed a high to intermediate resistivity in proximal and middle supratesticular zones; meanwhile, in the distal supratesticular zone, the values showed a low resistivity. These findings were in accordance with previously reported results in rams^10,19,20,25,26^. However, Hedia et al.^10^ reported that values of testicular blood flow including RI and PI were increased during summer, with a decline of the testicular volume in rams. This variation could be attributed to vascular resistance and/or testosterone level variation, and also to seasonal variation. Furthermore, no significant differences were found in Doppler indices values including RI and PI in both pre-pubertal and post pubertal rams^19,25^.

The findings of RI and PI values reported here may be explained by the fact that some parenchymal organs as testis normally have continuous blood flow, associated with gradually decreasing in the diastolic period and without reverse diastolic flow to insure constant blood perfusion for correct functioning^33^.

The wave forms of blood flow were biphasic and resistive in the proximal supratesticular zone of Assaf rams, and tended to be biphasic with intermediate resistivity in the middle supratesticular zone; meanwhile, distal supratesticular zone demonstrated monophasic and non-resistive wave form. In this regard and in contrast to our findings, previous studies reported monophasic non-resistive wave form in the supratesticular zone of testicular artery of rams, with no reference to the exact measurement location^10,25^. In stallion, the Doppler blood flow of the convoluted part of the testicular artery inside the spermatic cord revealed a resistive and biphasic character^34^. These differences of the wave forms might be attributed to variations of species, breed, testis position, season or blood vessels^1^. Thus, the supratesticular region of the testicular artery shows some differences in measurement of several blood flow parameters, propably related to convolutions and tortuosity^13^.

As detected here, doppler velocimetric values in different supratesticular zones showed a progressive and significant decline in flow velocity, in addition to pulsatility and vessel resistivity when entering the testis. Doppler velocities including PSV and EDV were highest in the proximal supratesticular zone of testicular artery of Assaf's rams; meanwhile, lowest values of these parameters were reported in the distal supratesticular zone of testicular artery. These findings were nearly similar to those observed by Camela et al.^19^. This fact, in turn, allows more contact time with the venous blood for heat exchange and gas exchange with the tissue^20^.

Furthermore, TABF and TABF rate exhibited a significant downward increment, where highest values were observed in the distal supratesticular zone. Interestingly, TABF and TABF rate parameters are used as reliable indicators for testicular perfusion, and they are more sensitive than other similar velocities or doppler indices for detection of small testicular perfusion changes^8^.

Morphometrical analysis of various layers of testicular artery wall revealed significant variations in thickness amongst different zones, where thicknesses of artery tunics were highest in the proximal supratesticular zone, and decreased distally. The highest thickness of tunica media thickness in addition to the lowest inner luminal diameter of testicular artery at the proximal supratesticular zone explains the fact of high blood resistance found normally in hemodynamic assessment during clinical approach of proximal testicular artery. Moreover, the blood resistivity decreases normally downward due to increased testicular artery prolongation and tortuosity, with reduced vascular endothelium thickness till entering the testis^13^.

The measured outer arterial diameters in histological sections were lower than those recorded by B-mode ultrasonography in the live animal at the same regions. These variations may be attributed to shrinkage effect of formalin fixation and tissue processing of testicular artery samples for histological analyses. Previous study revealed the decrease of canine blood vessel diameter by the histological processing^35^.

Tunica adventitia plays an essential role in healthy state of the arterial wall. It is consisting mainly of collagen fibers, and contains lymphatic tissues, nerves fibers and network of microvasculature known as vasa vasorum. In addition, a wide diversity of cells including macrophages, lymphocytes, dendritic cells, stem cell antigen progenitor cells, mast cells, and fibroblasts are found^36^.

Morphometrically, diameter of vasa vasorum was highest in the proximal supratesticular region of testicular artery, and decreased gradually distally toward the testis. These findings confirmed the fact that vascular wall thickness is an important factor for determination of vasa vasorum presence or absence^17,37^. Vasa vasorum is responsible for providing the vascular wall with nutritive and oxygen supplies^38^.

Smooth muscle cells (SMCs) are the main cell type of the tunica media. These cells regulate vascular tone and blood flow through dynamic cell contraction and relaxation. α-SMA is considered the first SMC marker to be expressed during embryogenesis, and at the latest is the most considerable protein in SMCs^36^. Here, the expression of α-SMA was higher in tunica media of proximal supratesticular zone of testicular artery than in the middle and distal supratesticular zones. This reduction in the expression may be attributed to the gradual decrease of tunica media thickness distally toward the testis.

Vimentin is a master intermediate filament protein in smooth muscle cells and tissues. It regulates the actin cytoskeleton in the smooth muscle^39^. Furthermore, vimentin is also expressed in endothelial cells of blood vessels, and that make it a good marker of microvascular distribution, since endothelial cells of blood vessels of all sizes, from capillaries to arteries, are strongly reactive^40^. Noticeably, vimentin showed immunoreactivity in the endothelial layer in all studied supratesticular zones. At proximal supratesticular zone, tunica media showed higher vimentin expression than in middle and distal supratesticular zones.

The obtained morphological and hemodynamic data showed to a great extent that the middle and distal supratesticular zones are nearly similar, where there were no significant differences between the two zones in about 60% of the obtained data. However, there were huge significant differences between the proximal and distal supratesticular zones in all obtained data. According to these findings, it is proposed that the middle supratesticular zone can be used as the best one to assess the hemodynamic of testicular artery in regular clinical diagnosis.

## Conclusion

The findings of this study can be summarized as follows: (1) The arterial diameter as shown by B-mode ultrasonography and histological analyses was widest in the distal supratesticular zone of testicular artery, followed by the middle and proximal zones; (2) Doppler indices RI and PI recorded high values in proximal supratesticular zone of testicular artery; however, in middle and distal ones, these values were lower. PSV showed the highest value at the proximal supratesticular zone of testicular artery, meanwhile, EDV showed a progressive increase distally toward the testis; (3) Thicknesses of tunica intima, media and adventitia were highest in the proximal supratesticular zone and decreased distally; (4) Vasa vasorum diameter was highest in the proximal supratesticular zone of testicular artery and decreased downward toward the testis; (5) Middle supratesticular zone of testicular artery was recommended as a best site to assess the hemodynamics of testicular artery in regular clinical diagnosis; (6) Alpha smooth muscle actin (α-SMA) and vimentin showed higher expression in the tunica media of proximal supratesticular zone than in the middle and distal ones. Findings reported here are considered baseline information for differentiation with pathological affections of the supratesticular region of testicular artery in this species. Future electron microscopic studies on different zones of the testicular artery within the supratesticular region should be done.

## Data Availability

The datasets analyzed during this study are available from the corresponding author on reasonable request.
